# Impact of Gentamicin Coadministration along with High Fructose Feeding on Progression of Renal Failure and Metabolic Syndrome in Sprague-Dawley Rats

**DOI:** 10.1155/2014/823879

**Published:** 2014-06-23

**Authors:** Zaid O. Ibraheem, Rusliza Basir, Ahmad Kh. Aljobory, Omar E. Ibrahim, Ajwad Alsumaidaee, Mun Fee Yam

**Affiliations:** ^1^Department of Pharmacology and Toxicology, School of Medicine and Health Science, Universiti Putra Malaysia, 47300 Serdang, Selangor, Malaysia; ^2^Department of Clinical Laboratory Science, Faculty of Pharmacy, Baghdad University, Baghdad, Iraq; ^3^Department of Anatomy, School of Medicine and Health Science, Universiti Putra Malaysia, 47300 Serdang, Selangor, Malaysia; ^4^Department of Veterinary Surgery, School of Veterinary Medicine, Universiti Putra Malaysia, 47300 Serdang, Selangor, Malaysia; ^5^Department of Pathology, School of Medicine and Health Science, Universiti Putra Malaysia, 47300 Serdang, Selangor, Malaysia; ^6^Department of Pathology, School of Dentistry, Universiti Technology MARA, 48600 Shah Alam, Selangor, Malaysia

## Abstract

The current study evaluates the impact of high fructose feeding in rat model of gentamicin induced nephrotoxicity. Sprague-Dawley rats weighing 180–200 g were randomized into four groups; (C) received standard rodents chow with free access to *ad libitum* drinking water for 8 weeks and was considered as control, (F) received standard rodents chow with free access to drinking water supplemented with 20% (W/V) fructose for the same abovementioned period, (FG) was fed as group F and was given 80 mg/kg (body weight)/day gentamicin sulphate intraperitoneally during the last 20 days of the feeding period, and (G) was given gentamicin as above and fed as group C. Renal function was assessed at the end of the treatment period through measuring serum creatinine, uric acid and albumin, creatinine clearance, absolute and fractional excretion of both sodium and potassium, twenty-four-hour urinary excretion of albumin, and renal histology. For metabolic syndrome assessment, fasting plasma glucose and insulin were measured and oral glucose tolerance test was performed throughout the treatment period. Results showed that gentamicin enhances progression of fructose induced metabolic syndrome. On the other hand, fructose pretreatment before gentamicin injection produced a comparable degree of renal dysfunction to those which were given fructose-free water but the picture of nephrotoxicity was somewhat altered as it was characterized by higher extent of glomerular congestion and protein urea. Overall, more vigilance is required when nephrotoxic drugs are prescribed for patients with fructose induced metabolic syndrome.

## 1. Introduction 

Gentamicin is an aminoglycoside antibiotic used to treat various Gram positive and Gram negative infections. Its nephrotoxic action is attributed to its aptitude to damage proximal convoluted tubules (PCT) and glomerular basement membrane [1]. Oxidative stress is the main causative factor of its nephrotoxicity [2, 3].

Ingestion of high amount of fructose in the form of sweet beverages or foodstuffs has a negative impact on the ability of the body to cope with various pathophysiological conditions [4]. In spite of that, they are used extensively in food industry due to their high sweetening capacity. Fructose induces metabolic changes characterized by hyperinsulinemia [5], hypertriglyceridemia, dyslipidemia, visceral obesity [6], and glucose intolerance [7].

Our study aimed to find the impact of combining of both fructose induced metabolic syndrome and gentamicin induced nephrotoxicity models on progression of both renal damage and deterioration of glucose homeostasis.

## 2. Materials and Methods

### 2.1. Animals and Diet

Male Sprague-Dawley rats, weighing 190 ± 4.7 g and obtained from animal house of Universiti Putra Malaysia, were used in the study. They were housed at the animal transit room with 4 animals per cage at room temperature with 12 : 12 h light-dark cycle. All the procedures were performed according to guidelines of the Malaysia Animal Ethics Committee for the use of animals in research.

**Figure 1:**
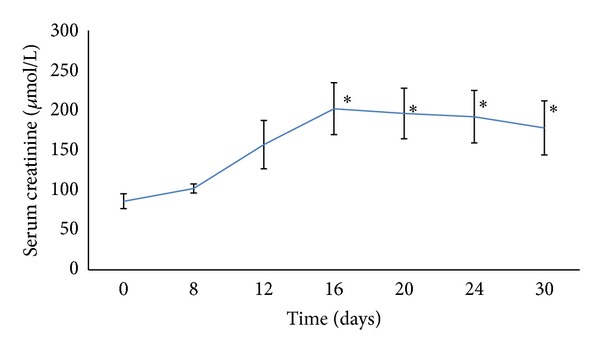
Serum creatinine in *μ*mol/L during gentamicin treatment during the preliminary study. “∗” indicates statistical significance (*P* < 0.05) as compared to day 0.

Time dependent effect for nephrotoxicity induction and the required sample size were estimated according to a preliminary study, in which 80 mg/kg/day gentamicin sulphate was injected intraperitoneally for 30 days with continuous monitoring of body weight and urine flow rate. Serum and urine samples were collected throughout the treatment period to assess progression of renal dysfunction. Maximum nephrotoxicity was obtained on day 16 and sustained up to the end of the treatment period (Figure 1). Then, twenty days were chosen as the treatment period to all the treatment protocols.

Before starting the experiment, rats were left for habituation and fed the standard commercial rodents chow with free access to water* ad libitum*. They were divided into four groups (10 animals each), such that C received standard rodents chow with free access to drinking water* ad libitum* for 8 weeks, F was fed with standard rodent chow with free access to 20% W/V fructose solution for the same abovementioned period, FG was fed like F group and received gentamicin sulphate intraperitoneally in a dose 80 mg/kg/day during the last 20 days of the feeding period, and G received the standard rodents chow and was given gentamicin sulphate as mentioned above. During the study, fructose solution was changed daily to avoid problems related to its fermentation.

### 2.2. Animals Monitoring

Body weight, water or fructose water, and food intake (corrected for spillage) were monitored continuously during the treatment period. Daily energy uptake was calculated from the amount of calories obtained from ingested fructose (4 Kcal/gm) and standard rodents chow (Table 1 shows composition and amount of calories in one gm of the standard rodents chow). Twenty-four hr urine and fasting tail vein serum samples were obtained on days 40 and 60 of the high fructose water treatment period which corresponds to days 0 and 20 of the gentamicin treatment period. At the end of the treatment period, I.V. glucose and insulin tolerance tests were performed while the animal was under phenobarbital anesthesia (60 mg/kg BW of phenobarbital was injected intraperitoneally). Eventually, animals were sacrificed, abdominal incision was performed, and each of epididymal, epirenal, and mesenteric fats was collected in order to calculate obesity index. Obesity index is the ratio of abdominal fat depot to total body weight [8].

### 2.3. F2 Group

Results of metabolic studies for F group showed that water uptake and urine flow rate were dropped after 8 weeks of fructose feeding. Gentamicin coadministration during the last 20 days of the feeding period (FG group) has raised urine flow rate. This polyurea urged FG group rats to drink more fructose enriched water making the total amount of fructose ingested by them higher than that of F group (Table 2). Metabolic study showed that total amount of daily energy uptake was comparable in both F and FG groups but the percentage of energy obtained from fructose was higher in FG group (Table 2). So accordingly, this suggests that any difference in the metabolic status of FG as compared to F is not merely attributed to the intervention of gentamicin therapy but it can be attributed to the higher amount of fructose that was ingested by FG group. To settle this confusion, we behooved to set another group of rats (F2 group). F2 group was fed with high fructose water in a mode similar to that of F group (high fructose water treated rats) till day 38, the time when gentamicin therapy had been commenced. Aftermath, in order to mimic fructose ingestion in FG group, the amount of fructose in the drinking water was gradually increased in F2 group throughout the next 20 days in an amount that make its ingestion comparable to what had been taken by FG. By this way, we could have overridden the impact that the exuberant fructose had been ingested by FG group and traced the precise effect of gentamicin on fructose induced metabolic disorders. The average amount of ingested fructose and water uptake for F and FG groups rats was measured weekly during the last 20 days of the fructose feeding period. We found that the amount of fructose ingested by each of the two groups in g/day was 3.33 ± 0.42, 3.42 ± 0.38, and 3.87 ± 0.41 for F group rats versus 3.74 ± 0.38, 5.05 ± 0.44, and 6.55 ± 0.43 for FG group rats within the first, second, and third trimesters of the abovementioned period, respectively, while water uptake in mL/day was 17.8 ± 1.3, 18.7 ± 2.1, and 19.2 ± 2.2 for F group and 21.4 ± 1.78, 25.5 ± 1.88, and 32.76 ± 1.9 for FG group within the first, second, and third trimesters of that period, respectively. In order to make fructose ingestion by F2 comparable to that of FG group, during each trimester of gentamicin treatment period, fructose solution containing the average amount of fructose ingested by FG group was prepared in a volume of water corresponding to the amount of water taken by F group during the correspondent period. According to this, we found that it was imperative to give fructose solution in a concentration of 20%, 26%, and 34% w/v during the first, second, and trimesters of the mentioned period.

### 2.4. Serum and Urine Biochemical Analyses

Serum was collected to measure urea, creatinine, albumin, total protein, oxidative stress markers (MDA and glutathione), uric acid, triglyceride, cholesterol, glucose, insulin, and both sodium and potassium concentrations. Urine samples were used to measure urine pH using pH meter and the twenty-four-hour excretion of sodium, potassium, and albumin. Creatinine clearance (Cr c) and both absolute and fractional excretions of the electrolytes were calculated using the conventional equations.

### 2.5. Renal Homogenate Study

One kidney from each rat was homogenized using 1 mL for each gram of kidney tissue of ice chilled 100 mM potassium chloride solution containing 0.3 mm EDTA using tissue homogenizer (Homogenizer MSE, England) [9]. TBARS (thiobarbituric acid reactive substance) level and glutathione were measured according to the established method.

### 2.6. Histology Study

Tissues were fixed using formalin solution. Then, they were subjected to the conventional histology procedures: procession, sectioning, staining, and slide mounting. Hematoxylin and eosin were used to stain the slides.

### 2.7. Data Analysis

Results were expressed as mean ± SEM. One-way ANOVA followed by Tukey's test was used for statistical analysis at 95% confidence level using students pack SPSS program version 16.

## 3. Results

### 3.1. Metabolic Study

Eight weeks of high fructose water uptake (F and F2 groups) resulted in a noticeable increase in the body weight, obesity index, and total amount of daily energy uptake with some statistical significance in comparison to control group (Table 1). On the other hand, I.P. injection of 80 mg/kg/day gentamicin sulphate to both high fructose and fructose-free water treated groups during the last 20 days of the treatment period did not produce any statistically significant change on these parameters as compared with their respective controls (Table 1). Furthermore, obesity index was a little bit higher in FG group as compared to F2 group (Table 2).

### 3.2. Metabolic Syndrome Parameters

**Figure 2:**
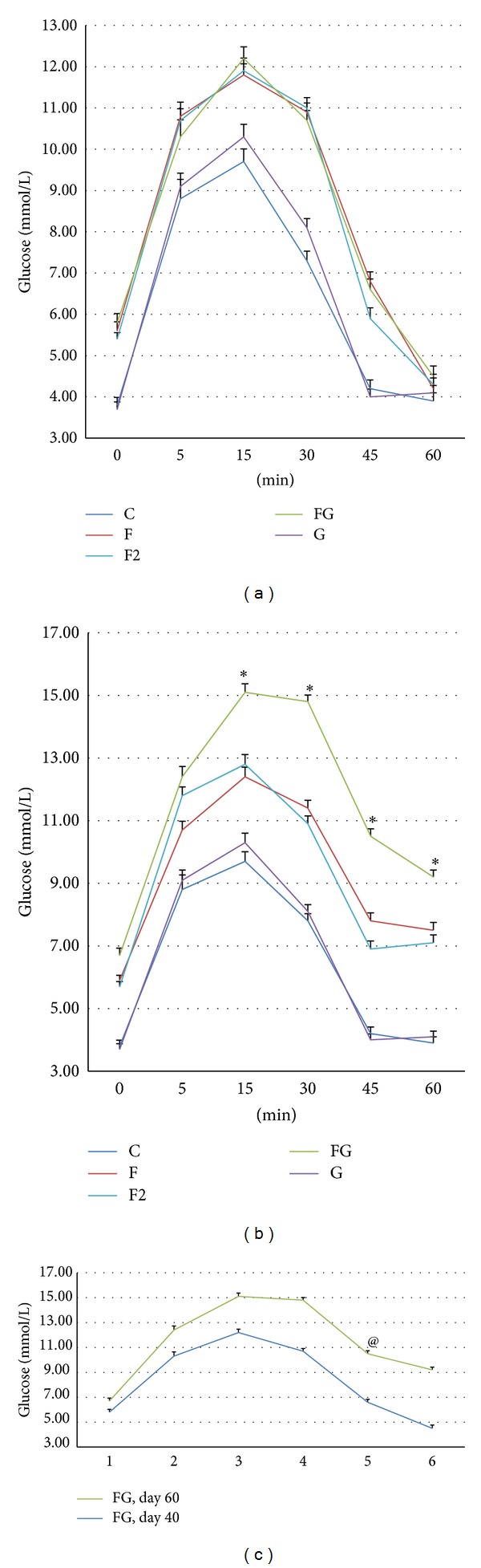
Oral glucose tolerance curve (OGTC) for C, F, FG, and G groups. Glucose level was measured at times 0, 5, 15, 30, and 45 min after giving 2 g/kg BW of glucose orally. (a) OGTC of the test groups on day 38 of the fructose treatment period or when gentamicin cotreatment was commenced. (b) OGTC of the test groups at the end of the treatment period. (c) OGTC of FG group on days 38 and 58 of fructose treatment or days 0 and 20 of gentamicin injection. Results are expressed as mean ± SEM. ∗, #, ‡,   and @ indicate statistical significance as compared to control, F, F2, FG, and G groups, respectively (*P* < 0.05). § indicates statistical significance as compared to G group (*P* < 0.05). @ indicates statistical significance as compared to day 0 of gentamicin treatment or day 36 of the fructose feeding.

Eight weeks of high fructose water ingestion (F and F2 groups) resulted in a condition characterized by metabolic syndrome as indicated by the prominent increase in fasting serum glucose, serum triglyceride, serum cholesterol, and serum insulin which showed some significance at some time points (Table 3, Figure 2). Furthermore, there was a noticeable shift in both oral glucose tolerance curves. Both F and F2 groups did not show any difference in these parameters indicating that the excessive fructose which was ingested by F2 group was not enough to change the metabolic status of the animal.

Gentamicin coadministration along with the fructose has worsened the metabolic syndrome as indicated by the higher increase in the abovementioned parameters in comparison to the high fructose water treated group (F2 group). On the other hand, gentamicin injection to rats given fructose-free water did not produce any effect on these parameters (Table 2, Figure 2).

### 3.3. Renal Failure Parameters

**Figure 3:**
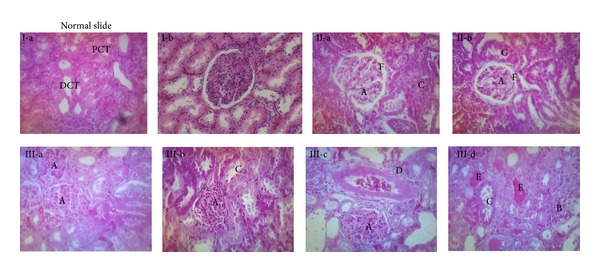
Kidney histology slides. Slides I-a and I-b belong to control (C), slides II-a and II-b belong to gentamicin treated group (G), and slides III-a, III-b, III-c, and III-D belong to the group that was treated with gentamicin along with high fructose water (FG). A indicates glomerular congestion and glomerular leucocytes infiltration. B indicates mesangial hyperproliferation. C indicates proximal convoluted tubule damage. D indicates interstitial inflammation and leucocytes infiltration. E indicates DCT ectasia by protein cast. F indicates glomerular basement membrane thickening.

Results of renal function study showed a comparable decline in renal function for FG as compared to G group except for presence of relatively stronger glomerular damage in FG group. According to Griffin classification of renal failure, mild to moderate degree of renal failure was observed in both of the renal failure groups as indicated by results of tubular and glomerular functions (Table 4) and histology study (Figure 3). Tubular dysfunction, as indicated by both absolute and fractional excretion of sodium, was comparable in both fructose fed and unfed rats but glomerular function and the potential of glomeruli to retain proteins were more deteriorated in the former group (Table 4). Histology slides revealed similar pattern of PCT (proximal convoluted tubule damage) in both FG and G rats but mesangial hyperproliferation, glomerular congestion, and distal convoluted tubules ectasia by protein cast were more pronounced in FG group as compared to G group (Table 6, Figure 3). Furthermore, 24 hr urinary excretion of protein was markedly elevated in the renal failure rats given high fructose water indicating that the glomerular damage was stronger in this group (Table 4).

High fructose water treatment for 8 weeks did not produce any significant change in the results of renal function assessment study except for a prominent increase in tubular function which is indicated by reduction of urine flow rate and both absolute and fractional excretions of potassium. Furthermore, the treatment causes a significant increase in uric acid level which was accompanied with a reduction in urine pH (Table 3).

Gentamicin injection to normal rats did not produce any change in uric acid level. On the other hand, surprisingly, gentamicin coadministration during the last 20 days of the high fructose water treatment period did not produce any change in uric acid level in comparison to the positive control (F2 group) in spite of more deterioration in the metabolic syndrome parameters after induction of renal failure (Table 4).

It is worthy to note that serum concentrations of both sodium and potassium were not affected in all the treatment groups. In spite of having a comparable absolute and fractional excretion of sodium in both of the fructose-free and high fructose water treated renal failure rats, potassium excretion was higher in the group treated with high fructose water as indicated by results of absolute and fractional excretion of potassium. There was a noticeable increase in the fractional excretion of potassium (Table 4).

### 3.4. Oxidative Stress

Oxidative stress assessment study in serum shows that fructose feeding produced some increase in serum oxidative stress as indicated by reduction of glutathione level and higher MDA production. Gentamicin coadministration along with fructose has raised the oxidative stress to its upmost level in comparison to that of the groups which are treated with gentamicin or high fructose water alone (Table 5).

Gentamicin administration produced a comparable increase in renal homogenate level of oxidative stress markers of rats fed with both normal and high fructose diet. Eight weeks of fructose feeding (F2 group) did not produce any change in the renal homogenate content of the oxidative stress markers (Table 5).

## 4. Discussion

Gentamicin is well documented as a nephrotoxic drug [3]. Pattern and lag time for its maximum nephrotoxicity rely on the animal status, feeding behavior, and the conditions of the experiment, such as the dosing profile [10]. Our preliminary study showed that nephrotoxicity increased progressively throughout the treatment period and reached its upmost level on day 16 and remained constant up to the end of the treatment period.

Glomerulonephritis (GN) and proximal convoluted tubules (PCT) tubulonephritis are the most characteristic features of gentamicin induced nephrotoxicity. The former is attributed to the aptitude of the drug for binding to glomerular basement membrane while the latter is due to its accumulation inside the lining epithelium of PCT. Gentamicin induces oxidative stress in the tubular epithelium resulting in loss of tubular function [1, 2]. Renal function study showed that daily injection of gentamicin intraperitoneally for 20 days to Sprague-Dawley rats has reduced glomerular filtration rate, increased serum urea and creatinine, decreased tubular reabsorption of both potassium and sodium, and produced histological changes characterized by mild PCT damage and interstitial infiltration along with glomerular congestion and hyalinization. This points out to development of tubular damage and drop in glomerular function.

High fructose water uptake for 8 weeks induced (F and F2 groups) metabolic changes characterized by metabolic syndrome as indicated by a noticeable increase in the obesity index, serum insulin, lipid profile, and the upward shifting of the glucose tolerance curve. The changes were comparable in both F and F2 indicating that the exuberant fructose that had been added to the drinking water in F2 group during the last 2 weeks of the treatment period was not enough to induce significant changes in metabolic syndrome parameters. Previous studies indicated that fructose can replenish glycolysis pathway extensively through passing phosphofructokinase step, the rate limiting step of the pathway. This reduces glucose uptake and triggers the uncontrolled flow of fructose derived carbons into lipogenesis pathway [11]. Furthermore, previous studies indicated that high fructose feeding inhibits tyrosine phosphorylation of insulin receptor after being exposed to insulin without affecting insulin binding to the receptors or their expression level [7]. This adversely affects the sequential flow of secondary signaling pathway that follows receptor activation. These observations were clearly seen in our results as lipid profile was obviously deteriorated after fructose treatment.

High fructose water ingestion produced changes in glucose homeostasis characterized by a prominent increase in basal insulin secretion and moderate change in insulin tolerance curve along with a pronounced change in glucose tolerance curve (Table 2, Figure 2). Unlike glucose, fructose induced metabolic syndrome is attributed mainly to the effect of fructose on insulin receptor phosphorylation while glucose induced metabolic syndrome is attributed to the higher insulin release and development of insulin resistance [12, 13]. Furthermore, fructose induced dyslipidemia and engorgement of fats in the visceral adipose tissue are also among the attributable factors that enhance fructose induced glucose intolerance [6]. Visceral fat does not merely act as a fat depot, but it acts also as an endocrine gland secreting inflammatory cytokines and macrophage chemotactant factors which can indirectly deteriorate insulin sensitivity and glucose tolerance [14]. Furthermore, it was found that fructose reduces insulin sensitivity through triggering release of the inflammatory cascade components, namely, tumor necrosis factor (TNF-*α*), c-Jun amino terminal kinase (JNK), nuclear factor-*κ*B, and STAT-3 (signal transducer and activator of transcription-3) [15–18]. On the other hand, it was found that fructose suppresses peroxisomes proliferation activated receptor-*α* (PPAR-*α*) activity in skeletal muscles and hepatocytes resulting in obviation of fatty acid oxidation and more lipid accumulation [19].

The inability of fructose to behave as glucose in eliciting a surge in leptin level made its impact on metabolic status more vicious [11]. Leptin is associated with satiety as it plays a role in regulation of food uptake. One study revealed that high fructose diet induces leptin resistance through inhibiting the hypothalamic signal transducer and activator of transcription-3 phosphorylation. It was found that this effect occurs before appearance of the other fructose high diet related phenomena, namely, body weight gain, hyperlipidemia, and glucose intolerance [20]. This interprets the noticeable increase in body weight, total daily energy uptake, and obesity index after fructose treatment.

Our results show that, in the high fructose water treated rats, urine flow rate, electrolyte excretion, and urine pH were significantly reduced in comparison to control. It is well known that hyperinsulinemia activates Na^+^/H^+^ pump in PCT resulting in increased uptake of Na^+^ and shedding more H^+^. This is accompanied by higher reabsorption of water, potassium, and bicarbonate [21, 22].

Urine acidification reduces uric acid seep with urine and augments hyperuricemia [23]. This interprets the significant development of hyperuricemia in the rats treated with the high fructose water. Hyperuricemia is culminated in enhancing progression of metabolic syndrome and oxidative stress. Uric acid increases oxidative stress in adipose tissue directly through activation of NADH oxidase enzyme and release of reactive oxygen species. On the other hand, uric acid can reduce endothelial capacity to produce nitric oxide [24].

High fructose feeding enhances release of methylglyoxal as a secondary intermediate product. The latter accumulates in tissues and is associated with higher emergence of reactive oxygen species and oxidative stress [25]. This augments insulin resistance in hepatocytes and skeletal muscles, lipogenesis, and engorgement of visceral adipose tissue with lipids [26]. Our results showed that there had been an increase in oxidative stress and reduction of total antioxidant status in the fructose fed rats.

Renal function study revealed that fructose feeding has changed the picture of gentamicin induced nephrotoxicity through producing a stronger glomerular damage. This was seen obviously through results of histology study and protein urea. Nevertheless, tubular function as indicated by tubular reabsorption of sodium was not affected. In spite of the observed higher glomerular damage in the renal failure group that received high fructose water in comparison to that which received* ad libitum* fructose free water, creatinine clearance was slightly affected. Protein urea was the most prominent biochemical change that accompanied this glomerular dysfunction. High fructose water treatment could not enhance tubular, although previous studies had pointed out the role of fructose induced metabolic syndrome in overactivation of the tubular function and increase of electrolytes reabsorption [25]. On the other hand, fructose can induce glomerular dysfunction through triggering more mesangial hyperproliferation and glomerular congestion [27]. Glomerular changes in metabolic syndrome are attributed to increased formation of advanced glycation end products AGE. Previously, it was found that fructose induced produces 10 times more AGEs than the glucose induced one. AGEs have the aptitude to cross-link glomerular membrane proteins causing the mentioned changes [27]. Our results showed higher proteinuria and DCT ectasia by protein cast in the group given gentamicin along with high fructose water as compared to that injected with gentamicin along with* ad libitum* feeding (Figure 3, Table 4). This is a natural consequence of the higher degree of glomerular damage in rats given gentamicin along with high fructose feeding.

Distal convoluted tubules (DCT) ectasia by protein cast is one of the prominent features of glomerular damage. It results from leakage of low g.m.wt proteins, like albumin. Proteins in the tubular filtrate precipitate the glycoproteins that line DCT (Tamm-Horsfall protein) resulting in creation of casts that block DCT. This observation was closely related to the observed protein urea in the renal failure rats fed with the high fructose treatment [28].

On the other hand, renal oxidative stress, as represented by measuring MDA (malondialdehyde) and glutathione content in renal homogenate, was significantly increased after gentamicin coadministration along with fructose treatment. In spite of its increase in the groups treated with gentamicin or fructose alone, the increase was higher in rats given the combined treatments. This may be attributed to the higher oxidative stress status or mesangial hyperproliferation that these rats experience.

Metabolic studies showed a significant increase in urine flow rate and water uptake in renal failure rats. Fructose uptake is directly proportional to the amount of water ingested in the groups treated with high fructose water. So gentamicin coadministration resulted in higher ingestion of fructose. In this group, the thrive to ingest standard rodents chow was decreased making the amount of energy uptake comparable to that of rats given* ad libitum* fructose-free water. Consequently, higher amount of fructose had been ingested by these animals. This can be controlled by limiting the amount of water uptake but this leads to water imbalance which is an important factor that affects the metabolic status. This case mimics the situation of drinking lots of fructose enriched beverages in patients who are under high dose of gentamicin treatment. This factor had raised a suggestion that metabolic abnormalities in the group given gentamicin along with high fructose water might have been attributed to the intervention of gentamicin or the higher amount of ingested fructose. Further studies are recommended to use high fructose chow diet instead of adding it in drinking water along with gentamicin so as to get a clearer picture of the direct intervention of gentamicin along with high fructose diet. This factor has behooved us to run F2 group which was designed to ingest the same amount of fructose taken by FG group.

Metabolic syndrome assessment study shows that the combined therapy was associated with higher deterioration in both glucose and lipid homeostasis as compared with fructose treatment model. This was indicated by results of fasting serum glucose, I.V. glucose and insulin tolerance tests, lipid profile, and obesity index in both F2 and FG groups. This change is more attributed to the plausible intervention of gentamicin on progression of fructose induced metabolic changes. Although FG rats consumed higher amount of fructose as compared to F rats, metabolic changes after gentamicin coadministration cannot be attributed to the higher fructose ingestion. Our results showed that there was not any significant change in progression of the metabolic disorders even after topping up the exuberant amount of fructose that was ingested by FG group. Gentamicin induced metabolic changes in the fructose induced metabolic syndrome rats might have been developed due to higher oxidative stress that gentamicin exerts as a part of its toxicity mechanism. This highlights the danger of providing more fructose sweetened beverages to patients who receive gentamicin therapy.

Interestingly, uric acid has increased significantly after fructose feeding. There was not any further increase in hyperuricemia in the combined model of fructose induced metabolic syndrome and gentamicin induced nephrotoxicity. Renal failure induction in FG group was supposed to worsen hyperuricemia. Its failure to do so suggests that mild to moderate gentamicin induced nephrotoxicity, which was observed in FG group, was not enough to further deteriorate uric acid homeostasis as compared to F or F2 group. On the other hand, it was suggested that gentamicin induced PCT damage reduces the tubular capacity to reabsorb HCO_3_^−^ which is required to push uric acid out [1]. This improves uric acid excretion and halts hyperuricemia progression. Gentamicin intervention along with fructose treatment did not intensify PCT damage but it opposed the conventional hyperinsulinemia induced PCT overactivity. Thence, it has reduced PCT aptitude to acidify urine and hindered uric acid exodus with urine.

In spite of the changes in urine flow rate, serum concentrations of both sodium and potassium were unaffected in all the treated groups. This may be due to the loss of equimolar amount of water along with the lost amount of sodium and the elaborate mechanism the body uses to maintain K^+^ concentration constant through regulation of cellular Na^+^-K^+^ pump and renal excretion of potassium [29]. Results of absolute and fractional excretion of potassium show a tendency of FG group kidneys to expel more K^+^ as compared to that which was given fructose-free water. During insulin resistance, K^+^ pumping into the cell ceases resulting in its leakage into the extracellular fluid [30]. Kidneys help the body to expel any exuberant K^+^ to maintain its level within narrow range [29]. On the other hand, gentamicin induced PCT damage results in seeping of more potassium into DCT and collecting tubules. The excessive sodium is reabsorbed there in replacement of potassium resulting in higher excretion of potassium with urine [31].

## 5. Conclusion

Feeding behavior affects the ability of the body to cope with different pathophysiological conditions and development of drugs toxicity. More precaution measures should be taken when drugs are prescribed for metabolic syndrome patients as they may enhance progression of metabolic syndrome and metabolic syndrome enhances progression of the drug toxicity.

## Figures and Tables

**Table 1:** Food and calories composition of the standard rodent chow.

Constituents	Standard chow diet	
	Wt. (g)	Calories
Protein	0.20	0.8
Carbohydrates	0.77	2.38
Fat	0.03	0.12
Cholesterol	—	—
Cholic acid	—	—
Calcium	0.012	—
Phosphate	0.012	—
Sodium	0.021	—
Potassium	0.007	—

Total	1	3.3

**Table 2:** Metabolic study parameters.

Parameter	Time	Control	F	F2	FG	G
Percent of weekly increment in body weight	W1	3.3 ± 0.26	4.2 ± 0.39	4.3 ± 0.34∗	4.3 ± 0.34∗	3.6 ± 0.31
	W2	3.6 ± 0.28	4.4 ± 0.31∗	4.7 ± 0.27∗	4.7 ± 0.27∗	3.0 ± 0.34
	W3	3.7 ± 0.29	4.3 ± 0.33∗	5.1 ± 0.23	4.5 ± 0.26	3.1 ± 0.29

Energy uptake Kcal/day/100 g (BW)	W1	23 ± 1.4	26 ± 1.2	26 ± 1.2	26 ± 1.2	23 ± 1.4
	W2	21 ± 1.3	28 ± 1.6	33.2 ± 1.5^@^	32.2 ± 1.4^@^	18 ± 1.1^#,$^
	W3	20 ± 0.9	27 ± 1.6	35.1 ± 1.9∗	33.9 ± 1.7∗	19 ± 1.2

Energy taken from fructose Kcal/day/100 g (BW)	W1	0	9.31 ± 0.39	10.18 ± 0.38	10.18 ± 0.38	0
	W2	0	8.92 ± 0.38	14.64 ± 0.68^‡^	15.24 ± 0.68^‡^	0
	W3	0	8.78 ± 0.26	15.25 ± 0.54^‡^	16.15 ± 0.54^‡^	0

Water uptake *μ*L/min	W1	15.2 ± 1	12.8 ± 0.6	12.4 ± 0.53	11.4 ± 0.48	15.3 ± 1
	W2	14.3 ± 1.2	13.6 ± 0.8	13.4.4 ± 1.93	22.4 ± 1.32^∗,‡,#,^	21.6 ± 2.95
	W3	15.4 ± 1.1	12.4 ± 0.65	11.1 ± 1.8	23.1 ± 1.9^∗,‡,#^	24 ± 2.1^∗,‡,#,^

UFR *μ*L/min/100 g (BW)	W1	2.18 ± 0.2	1.70 ± 0.1	1.53 ± 0.1	1.44 ± 0.1∗	—
	W2	2.09 ± 0.18	1.62 ± 0.10^$^	1.49 ± 0.12^$^	5.42 ± 0.5^∗,‡,#^	4.30 ± 0.60
	W3	2.16 ± 0.12	1.55 ± 0.08^$^	1.43 ± 0.06^∗$^	4.95 ± 0.7^∗,‡,#,^	4.06 ± 0.27∗

Percentage of increment of body weight was performed weakly while calories and water uptake and urine flow rate were measured in the 1st, 2nd, and 3rd weeks of gentamicin treatment which correspond to the last 3 weeks of the high fructose water treatment period. Results are expressed in mean ± SEM. ∗, ‡, #, $, and @ indicate statistical significance as compared to control, F, F2, FG, and G groups, respectively (*P* < 0.05).

**Table 3:** Metabolic syndrome assessment parameters.

Parameter	Time	Control	F	F2	FG	G
F glu mmol/L	d0	3.8 ± 0.18	5.6 ± 0.25∗	5.5 ± 0.30∗	5.8 ± 0.22∗	3.7 ± 0.19
	d20	4.4 ± 0.18	5.9 ± 0.16∗	6.1 ± 0.23∗	6.7 ± 0.19∗	4.2 ± 0.18

Serum cholesterol mmol/L	d0	0.87 ± 0.08	1.17 ± 0.05	1.18 ± 0.09	1.24 ± 0.09	0.84 ± 0.08
	d20	0.92 ± 0.07	1.19 ± 0.07	1.24 ± 0.06	1.37 ± 0.13∗	0.77 ± 0.09

Serum triglyceride mmol/L	d0	0.65 ± 0.1	1.27 ± 0.15∗	1.30 ± 0.12∗	1.32 ± 0.11∗	0.84 ± 0.08
	d20	0.70 ± 0.13	1.24 ± 0.10∗	1.29 ± 0.14∗	1.67 ± 0.14∗	0.77 ± 0.09

Serum insulin *μ* I.U/mL	d0	24.2 ± 1.9	23.3 ± 1.9	25.2 ± 1.7	25.2 ± 1.7	26.4 ± 1.9
	d20	26.2 ± 2.1	29.4 ± 2.3	31.3 ± 1.9∗	38.3 ± 1.9∗	24.1 ± 2.1

Obesity index	d20	2.04 ± 0.13	2.75 ± 0.22^∗@^	2.74 ± 0.21^∗@^	3.14 ± 0.21^∗@^	2 ± 0.16^‡,#,$^

Serum parameters measurements were taken on days 0 and 20 of gentamicin therapy which correspond to the 38th and the 58th days of the high fructose water treatment period. Obesity index was measured at the end of the treatment period. Results are expressed in mean ± SEM. ∗, ‡, #, $, and @ indicate statistical significance as compared to control, F, F2, FG, and G groups, respectively (*P* < 0.05).

**Table 4:** Renal function assessment parameters (tubular and glomerular functions) along with uric acid level and kidney index (ratio of kidney weight to total body weight).

Parameter	Time	Control	F2	FG	G
Glomerular function					
Serum urea mmol/L	d0	18 ± 2.9	22.1 ± 3.4	26 ± 2.9	17.8 ± 2.8
	d20	21 ± 2.8	32.2 ± 3.5	52.8 ± 2	24.7 ± 2.4
Serum creatinine	d0	79 ± 5.1	88 ± 4.1	78.6 ± 4	—
	d20	86 ± 5.8^@,$^	91 ± 4.8^@,$^	199 ± 11^∗,‡^	182 ± 12^∗,‡^
Creatinine clearance mL/min/100 g (B.W)	d0	0.16 ± 0.02	0.16 ± 0.03	—	—
	d20	0.14 ± 0.02^@,$^	0.12 ± 0.01^@,$^	0.075 ± 0.029^∗,‡^	0.09 ± 0.03^∗,‡^
Urinary protein excretion mg/hr	d0	0.055 ± 0.008	0.045 ± 0.007	0.051 ± 0.006	0.049 ± 0.007
	d20	0.052 ± 0.006	0.039 ± 0.007	0.094 ± 0.009^∗@‡^	0.059 ± 0.007
Serum sodium and potassium					
Serum Na^+^	d0	136 ± 2.1	134 ± 2.1	135 ± 1.4	137 ± 1.8
	d20	139 ± 1.8	141 ± 2.8	133 ± 2.3	135 ± 1.7
Serum K^+^	d0	5.7 ± 0.2	5.6 ± 0.22	5.6 ± 0.18	5.5 ± 0.21
	d20	5.6 ± 0.23	5.9 ± 0.24	5.3 ± 0.27	5.7 ± 0.23
Tubular function					
Abs. Na^+^ excretion ×10^−3^ mmol/hr	d0	17 ± 1.60	11 ± 1.00∗	9 ± 1.10∗	—
	d20	18 ± 1.70	10 ± 0.90^@^	34 ± 5.20	34 ± 2.80^∗,‡^
UFR *μ*L/min/100 g (BW)	d0	2.18 ± 0.2	1.70 ± 0.1	1.53 ± 0.1	1.44 ± 0.1∗
	d20	2.16 ± 0.12	1.55 ± 0.08	1.43 ± 0.06∗	4.95 ± 0.76^∗,#^
Abs. K^+^ excretion mmol/hr	d0	0.07 ± 0.01	0.05 ± 0.01∗	0.04 ± 0.00∗	—
	d20	0.07 ± 0.01	0.05 ± 0.01^$^	0.27 ± 0.03^∗,‡^	0.17 ± 0.015∗
FE Na^+^	d0	0.51 ± 0.06	0.37 ± 0.034	0.33 ± 0.031	—
	d20	0.49 ± 0.03	0.39 ± 0.021^$@^	2.33 ± 0.12^∗‡^	2.03 ± 0.155^∗‡^
FE K^+^	d0	61 ± 5.94	39 ± 3.7	48 ± 6.94	—
	d20	67 ± 6.87^$^	49 ± 3.21^@$^	396 ± 44.5^∗,#,@^	195 ± 33.77^∗‡^
Kidney index					
Kidney index	d20	0.67 ± 0.01	0.67 ± 0.02	0.69 ± 0.05	0.7 ± 0.03
Uric acid					
Uric acid *μ*mol/L	d0	11.3 ± 1.37	17.1 ± 1.44^∗@^	16.4 ± 1.38^∗@^	11.8 ± 1.35^‡,$^
	d20	2.7 ± 1.32	19.6 ± 1.43∗	13.9 ± 1.40	12.9 ± 1.29
Urine pH	d0	6.7 ± 0.34	5.3 ± 0.54	5.5 ± 0.61	6.4 ± 0.29
	d20	6.9 ± 0.33	4.9 ± 0.56	5.9 ± 0.55	7.2 ± 0.2.8

The measurements were taken on days 0 and 20 which correspond to 38th and 58th days of the high fructose water treatment period. Kidney index was measured at the end of the treatment period. Results are expressed in mean ± SEM. ∗, ‡, $, and @ indicate statistical significance as compared to control, F2, FG, and G groups, respectively (*P* < 0.05).

**Table 5:** Oxidative stress parameters (malondialdehyde (MDA) and glutathione) in both serum and renal homogenates.

Parameter	Control	F2	FG	G
Oxidative stress in serum at the end of the treatment period (day 24)				
Serum MDA nmol/mL	2.735 ± 0.16	3.46 ± 0.32	5.783 ± 0.31∗	2.86 ± 0.19
Serum glutathione (GSH) mL/L	167 ± 5.23	145 ± 5.8	103 ± 4.9∗	159 ± 6.3
Oxidative stress in kidney homogenate at the end of the treatment period				
MDA in nmol/g kidney tissue	65.4 ± 2.3	67.1 ± 2.6	83.2 ± 3.7	79.4 ± 5.8
GSH glutathione mg/g kidney tissue	98.5 ± 3.1	97.5 ± 2.8	89.5 ± 3.2	91.2 ± 2.6

The measurements were done at the end of the treatment period. Results are expressed as mean ± S.E.M. ∗, ‡, $, and @ indicate statistical significance as compared to control, F2, FG, and G groups, respectively (*P* < 0.05).

**Table 6:** Histological changes grading for the FG and G groups at the end of the treatment period.

Histological change/rat	Glomerular congestion	Mesangial hyperproliferation	PCT damage	Interstitial PMNs infiltration	DCT ectasia by protein cast	Final decision
FG 1	++	++	+	Nill	+	Moderate
FG 2	+	+	+	+	Nill	Mild
FG 3	++	++	+	+	++	Moderate
FG 4	Nill	Nill	Nill	Nill	Nill	Normal
FG 5	+	+	+	Nill	+	Mild to moderate
FG 6	+	+	+	+	+	Mild to moderate
FG 7	++	+	Nill	+	++	Moderate
FG 8	+	+	Nill	+	Nill	Mild

G1	+	Nill	Minimal	Nill	Nill	Very mild
G2	Nill	Nill	+	Nill	Nill	Mild
G3	+	Nill	++	Nill	Nill	Mild to moderate
G4	Nill	Nill	+	Nill	Nill	Mild
G5	Nill	Nill	Nill	Nill	Nill	Normal
G6	+	Nill	Nill	Nill	Nill	Mild
G7	+	Nill	+	Nill	Nill	Mild
G8	+	Nill	+	Nill	+	Mild
G9	+	Nill	++	+	+	Moderate
G10	+	Nill	++	+	Nill	Moderate
